# THE ROLE OF PLATELET ENERGY METABOLISM IN PAIN AFTER WALKING IN BLACK ADULTS WITH KNEE OSTEOARTHRITIS

**DOI:** 10.1093/geroni/igac059.2449

**Published:** 2022-12-20

**Authors:** Jennifer Klinedinst, Weiliang Huang, Maureen Kane, Gary Fiskum, Apruva Borcar, Parisa Rangghran, Susan Dorsey

**Affiliations:** University of Maryland School of Nursing, Baltimore, Maryland, United States; School of Pharmacy, Baltimore, Maryland, United States; University of Maryland Baltimore, Baltimore, Maryland, United States; University of Maryland School of Medicine, Baltimore, Maryland, United States; University of Maryland School of Medicine, Baltimore, Maryland, United States; University of Maryland School of Medicine, Baltimore, Maryland, United States; University Of Maryland School of Nursing, Baltimore, Maryland, United States

## Abstract

Nearly 30% of adults aged 60 or older suffer from knee osteoarthritis (KOA) that causes significant pain and disability. Walking is considered a “gold standard” treatment option for reducing KOA pain and maintaining joint mobility. However, pragmatic trials have shown that walking increases pain for some and relieves pain for others. The mechanism by which walking is helpful for KOA pain is unclear. The purpose of this study was to gain a better understanding of the mechanisms underlying walking for knee pain. We conducted a pre-test/post-test study using quantitative sensory testing to measure pressure pain sensitivity at the knee and examined protein signatures and measured energy metabolism in platelets in six adults with KOA before and after six weeks of walking three days/week at 100 steps/minute. All participants identified as Black/African American, five were female, average age 57±5.8. Pressure pain sensitivity increased for three participants and decreased for three participants. Protein signatures among KOA participants indicated differences in immune and energy metabolism pathways. Proteins in the energy metabolism pathways were significantly downregulated after walking in participants whose pain increased compared to participants whose pain decreased. Platelet energy metabolism was also lower among participants whose pain increased as compared to participants whose pain decreased. One goal of developing individualized interventions for KOA pain is to elucidate the mechanisms by which self-management interventions impact pain. The addition of therapies that target cellular energy metabolism may lower pain with walking among Black adults with KOA.

